# Personalised selection of medication for newly diagnosed adult epilepsy: study protocol of a first-in-class, double-blind, randomised controlled trial

**DOI:** 10.1136/bmjopen-2024-086607

**Published:** 2025-04-05

**Authors:** Daniel Thom, Richard Shek-Kwan Chang, Natasha A Lannin, Zanfina Ademi, Zongyuan Ge, David Reutens, Terence O’Brien, Wendyl D’Souza, Piero Perucca, Sandra Reeder, Armin Nikpour, Chong Wong, Michelle Kiley, Jacqui-Lyn Saw, John-Paul Nicolo, Udaya Seneviratne, Patrick Carney, Dean Jones, Ernest Somerville, Clare Stapleton, Emma Foster, Lata Vadlamudi, David N Vaughan, James Lee, Tania Farrar, Mark Howard, Robert Sparrow, Zhibin Chen, Patrick Kwan

**Affiliations:** 1 Department of Neuroscience, School of Translational Medicine, Monash University, Melbourne, Victoria, Australia; 2 Department of Neurology, Alfred Hospital, Melbourne, Victoria, Australia; 3 Allied Health Directorate, Alfred Health, Melbourne, Victoria, Australia; 4 Health Economics and Policy Evaluation Research Group, Monash Institute of Pharmaceutical Sciences Centre for Medicine Use and Safety, Parkville, Victoria, Australia; 5 Department of Data Science and AI, Monash University, Clayton, Victoria, Australia; 6 Department of Neurology, Royal Brisbane and Women’s Hospital, Herston, Queensland, Australia; 7 Department of Neurology, Alfred Health, Melbourne, Victoria, Australia; 8 Department of Neurology, St Vincent’s Hospital Melbourne Pty Ltd, Fitzroy, Victoria, Australia; 9 Bladin-Berkovic Comprehensive Epilepsy Program, Department of Neurology, Austin Health, Heidelberg, Victoria, Australia; 10 Epilepsy Research Centre, Department of Medicine (Austin Health), The University of Melbourne, Melbourne, Victoria, Australia; 11 Department of Neurology, Royal Prince Alfred Hospital, Camperdown, New South Wales, Australia; 12 The Brain and Spine Centre, Westmead Hospital, Westmead, New South Wales, Australia; 13 Department of Neurology, Royal Adelaide Hospital, Adelaide, South Australia, Australia; 14 Department of Neurology, Queen Elizabeth Hospital, Adelaide, South Australia, Australia; 15 Department of Neurology, Royal Perth Hospital, Perth, Western Australia, Australia; 16 Department of Neurology, The Royal Melbourne Hospital, Melbourne, Victoria, Australia; 17 Department of Neurology, Monash Medical Centre, Clayton, Victoria, Australia; 18 Department of Neurosciences and Eastern Health Clinical School, Eastern Health, Box Hill, Victoria, Australia; 19 Department of Neurology, Royal Hobart Hospital, Hobart, Tasmania, Australia; 20 Department of Neurology, Prince of Wales Hospital, Sydney, New South Wales, Australia; 21 Lived Experience Expert, Sydney, New South Wales, Australia; 22 The University of Queensland, Brisbane, Queensland, Australia; 23 Florey Institute of Neuroscience and Mental Health, Heidelberg, Victoria, Australia; 24 Department of Neurology, Royal North Shore Hospital, St Leonards, New South Wales, Australia; 25 Department of Philosophy, Monash University, Melbourne, Victoria, Australia; 26 Department of Philosophy, Monash University, Clayton, Victoria, Australia; 27 Department of Neurology, The Royal Melbourne Hospital, Parkville, Victoria, Australia

**Keywords:** Artificial Intelligence, Machine Learning, Epilepsy, Clinical Trial

## Abstract

**Introduction:**

Selection of antiseizure medications (ASMs) for newly diagnosed epilepsy remains largely a trial-and-error process. We have developed a machine learning (ML) model using retrospective data collected from five international cohorts that predicts response to different ASMs as the initial treatment for individual adults with new-onset epilepsy. This study aims to prospectively evaluate this model in Australia using a randomised controlled trial design.

**Methods and analysis:**

At least 234 adult patients with newly diagnosed epilepsy will be recruited from 14 centres in Australia. Patients will be randomised 1:1 to the ML group or usual care group. The ML group will receive the ASM recommended by the model unless it is considered contraindicated by the neurologist. The usual care group will receive the ASM selected by the neurologist alone. Both the patient and neurologists conducting the follow-up will be blinded to the group assignment. Both groups will be followed up for 52 weeks to assess treatment outcomes. Additional information on adverse events, quality of life, mood and use of healthcare services and productivity will be collected using validated questionnaires. Acceptability of the model will also be assessed.

The primary outcome will be the proportion of participants who achieve seizure-freedom (defined as no seizures during the 12-month follow-up period) while taking the initially prescribed ASM. Secondary outcomes include time to treatment failure, time to first seizure after randomisation, changes in mood assessment score and quality of life score, direct healthcare costs, and loss of productivity during the treatment period.

This trial will provide class I evidence for the effectiveness of a ML model as a decision support tool for neurologists to select the first ASM for adults with newly diagnosed epilepsy.

**Ethics and dissemination:**

This study is approved by the Alfred Health Human Research Ethics Committee (Project 130/23). Findings will be presented in academic conferences and submitted to peer-reviewed journals for publication.

**Trial registration number:**

ACTRN12623000209695.

**STRENGTHS AND LIMITATIONS OF THIS STUDY**

- Multisite, prospective, double-blind, randomised, controlled trial of a machine learning model for antiseizure medication (ASM) selection for newly diagnosed epilepsy.
- Besides measuring the cost-effectiveness of applying the machine learning model, the acceptance of machine learning-assisted clinical decision support will be explored among different stakeholders, including both patients and neurologists.
- The trial confines the choice of drug to seven commonly prescribed ASMs, limiting the flexibility of neurologists who may have prescription preference outside the repertoire.
- The inclusion criteria of aged 18–67 specifically exclude the older adult epilepsy cohort who have a high prevalence of epilepsy along with distinct health and social needs that could be alleviated by optimised initial ASM selection.
- The model is used both on patients with generalised epilepsy or focal epilepsy, planned subgroup analysis is limited to these two epilepsy types and does not include more specific aetiologies.

## Introduction

 Epilepsy is a common chronic, serious neurological disorder that affects all age groups and ethnicities worldwide. Over 50 million people are living with epilepsy, accounting for 13 million disability-adjusted life-years.^1^ Antiseizure medications (ASMs) are the mainstay of epilepsy therapy, with the primary goal of achieving the best possible quality of life by maximising seizure control and minimising adverse effects.^2 3^ For a newly diagnosed patient, it is recommended to start an ASM as monotherapy. In the current treatment paradigm, a clinician selects an ASM according to the patient’s characteristics including demographics, epilepsy type/syndrome, comorbidities, while also considering the ASM efficacy spectrum and tolerability/safety profile. There are more than 20 ASMs available; among those approved for use as monotherapy, none has shown overwhelming superiority in terms of efficacy and tolerability.^4^ Choosing the ideal ASM for an individual can be a subjective and even biased process. There is no reliable biomarker to predict which ASM will be the most effective for an individual patient. As a result, people with epilepsy can undergo multiple trials of various medication regimens before attaining seizure freedom.^5^ The consequence can be years of poor quality of life,^6^ productivity loss^7^ and even increased risk of mortality.^8^

Failure of the first ASM is the strongest predictor of long-term drug resistance and poor health outcomes.^9 10^ Choosing the first ASM is arguably the most important decision to be made in the management of newly diagnosed epilepsy from the perspectives of both clinical outcome and the patient’s preference. Hence, it would be of great clinical value if the most effective one could be prescribed at the outset instead of going through the current trial-and-error process.

Machine learning (ML) is a subfield of artificial intelligence (AI). It has the advantages in recognising hidden patterns in input variables and making meaningful predictions. Our group has recently developed a deep learning model, a type of ML model with multiple layers, that used 16 structured clinical variables to predict the effectiveness of ASMs in adults with newly diagnosed epilepsy.^11^ The model was trained and tested on five independent cohorts from four countries (UK, Malaysia, Australia, and China) totalling 1798 patients. It was trained to predict and rank the likelihood of treatment success (seizure freedom at 12 months without intolerable adverse effects) of each of the seven predefined ASMs (which include the most used ASMs in Australia) as the first monotherapy for an individual patient. This ability is illustrated in 100 randomly selected individuals from the pooled cohort in the online supplemental figure. Using the observed response to the prescribed ASM as the ‘ground truth’, our model outperformed five other traditional ML and deep learning algorithms in the prediction.

We have improved this model by applying natural language processing (NLP) to extract information from the text of electroencephalography (EEG) and MRI brain reports. The NLP output is incorporated as supplementary input alongside the structured clinical variables to develop a multimodality ML model. Consideration was made regarding the inclusion of sleep EEG reports, while they are not precluded from use in place of a routine EEG, for pragmatic reasons they have not been made mandatory. The improved model was reoptimised using datasets from Australian and international cohorts. Ultimately, the effectiveness of applying such a model in clinical practice would require prospective evaluation.

This article describes the protocol of a randomised controlled trial (RCT) that aims to evaluate the clinical effectiveness of the multimodality ML model in selecting the first ASM for adults with newly diagnosed epilepsy. The trial will also assess the cost-effectiveness and acceptance of the model to patients and neurologists. This protocol follows the Standard Protocol Items: Recommendations for Interventional Trials-AI extension guidelines for clinical trials of interventions involving AI.^12^

## Methods and analysis

### Study design and setting

This is a multicentre, randomised, controlled, patient and assessor blinded trial conducted in Australia. It is considered ‘first-in-class’ as the first RCT of an ML model in epilepsy management. Adults of working age with newly diagnosed epilepsy will be referred to the study by their treating clinicians. Participants will be recruited from 14 epilepsy centres across 5 Australian states from 14 February 2024 to 13 February 2026.

### Intervention

The intervention in this study is known as EpiMD, a clinical decision support software developed to assist the neurologist in selecting the first ASM. EpiMD is a multimodality ML model using the deep learning technique known as multilayer perceptron. A multilayer perceptron is focused on mapping the relationships between different variables.^13^ EpiMD includes clinical variables, as well as annotated EEG and MRI brain reports that have undergone NLP. Using these inputs, EpiMD will recommend the ASM monotherapy (from a group of seven possible choices) with which the participant is predicted to have the highest probability of achieving seizure freedom for the 12 months following ASM commencement. After consultation with the site investigators, the implementation of EpiMD will include a filter to prevent the recommendation of sodium valproate for women of childbearing age (defined in this study as between 18 and 45 years of age) owing to the medication’s teratogenic risk. No other filtering will be applied.

### Eligibility criteria

Inclusion criteria for participants are the following: aged 18–67 years, capacity to self-consent, fluent in English, diagnosis of epilepsy consistent with the International League Against Epilepsy (ILAE) definition.^14^ This is operationally defined as having had at least two seizures within the past 12 months, or one seizure within the past 6 months and epileptiform discharges on EEG, or presence of epileptogenic lesion on CT or MRI. Routine EEG and MRI brain done within 2 years prior to randomisation is also required. The routine EEG can (but is not specifically required to) be replaced with a sleep EEG.

Exclusion criteria for participants are the following: previously treated epilepsy or current ASM use for more than 14 days, pregnant or breastfeeding, current substance abuse disorder that may affect treatment adherence/response, inability to undergo EEG/MRI brain due to contraindication, presence of functional seizures, presence of progressive central nervous system disease, serious hepatic or renal disease, or terminal cancer that would affect assessment of response to ASMs.

### Recruitment procedures

**Figure 1:**
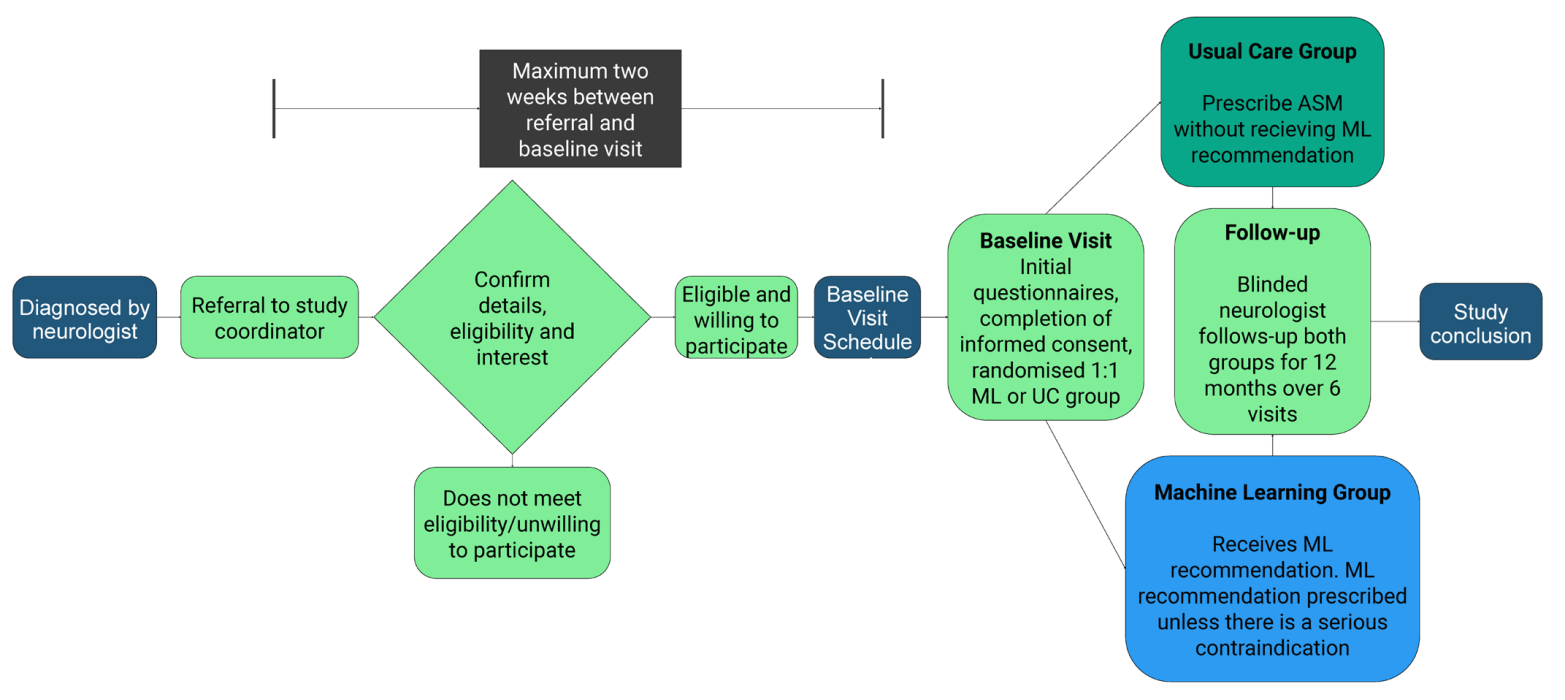
Study recruitment pathway. Study recruitment pathway showing sequencing of referral, screening and consenting procedures, in addition to randomisation into the two study groups, before merging into an identical follow-up pathway. ASM, antiseizure medication; ML, machine learning; UC, usual care.

Figure 1 shows the recruitment pathway. Treating neurologists will prospectively identify participants from outpatient clinics and inpatient wards using the eligibility criteria. Participants who meet the criteria will be referred to the study coordinator, who will contact the patient to explain all details of the study in addition to providing a copy of the patient information and consent form (PICF). If the participant is interested in taking part in the study, a baseline study visit in the presence of a neurologist acting as an investigator will be conducted via video link or in-person within 2 weeks of the initial referral.

At the baseline visit, if all the eligibility criteria are fulfilled, the PICF will be signed by the participant and investigator. The baseline questionnaires will be completed by the participant, guided by the neurologist and study coordinator, to collect clinical variables alongside diagnostic test reports from the patient electronic medical record (EMR). Additionally, the content and utilisation of both the seizure diary and the healthcare use diary that will be completed at follow-up visits will be explained to the participant. Study data are collected and managed using Research Electronic Data Capture (REDCap) tool hosted and managed by Helix (Monash University).^15 16^ REDCap is a secure, web-based software platform designed to support data capture for research studies. It provides an intuitive interface for validated data capture and audit trails for tracking data manipulation and export procedures. REDCap will be used to automate the export procedures for data downloads to common statistical packages and the procedures for data integration and interoperability with external sources. The trial REDCap is updated anytime the model is utilised, and therefore, is suitable to meet our obligations regarding data management and site monitoring.

### Randomisation and ASM prescription

The participant will be randomised 1:1 into either the experimental (ML group) or the control group (usual care group) using individual randomisation centralised and in blocks that are stratified by both seizure type (focal vs generalised onset) and study site. In the experimental group, EpiMD will be run using the information provided and respond with the ASM recommendation. The investigator will prescribe the recommended ASM unless it is considered to be contraindicated due to safety reasons. In this case, the investigator will prescribe a different ASM and provide their reasoning for doing so. In the control group, the recommendation by the ML model will not be revealed, and the investigator will prescribe an ASM based only on their clinical judgement.

Any out-of-pocket costs associated with new generation ASMs used as monotherapy that are not covered by the Pharmaceutical Benefits Scheme will be reimbursed to the participants. ASM prescription will be open label for both groups. Guidance regarding the rate of titration, initial maintenance dose and any subsequent increments or decrements will be made available to the prescribing clinician. If the patient is taking an ASM prior to randomisation, the drug will be ceased over 2–4 weeks under the supervision of the treating neurologist.

### Blinding

Both the participant receiving treatment and the neurologist assessing outcomes at follow-up visits will be blinded as to which group the participant has been assigned; however, neither the patient nor the treating neurologist will be blinded to the ASM that is being taken. Blinding will be maintained through either of two methods. In method 1, the referring neurologist will act as the investigator at the baseline visit and prescribe the ASM, and different neurologist(s) will perform all subsequent follow-up. In method 2, a neurologist different from the referring one will prescribe the ASM at the baseline visit, and the referring neurologist will perform all subsequent follow-up. This allows the continuity of care at participating epilepsy clinics to be maintained.

### Follow-up procedures

Prior to a scheduled study visit, participants will be sent a REDCap survey invitation to the email they have provided. This email includes a link to a series of surveys associated with the upcoming visit as listed in table 1. These will be limited to only the seizure diary and the Liverpool Adverse Events Profile (LAEP) for the minor follow-up visits (8 weeks, 16 weeks and 40 weeks). For the major visits (28 weeks and 52 weeks), the patient will also complete surveys on mood, quality of life, healthcare utilisation and productivity. During the follow-up visits, the surveys will be reviewed for completion and discussed with the participant. If assistance is required, the participants can opt to complete the surveys together with the neurologist and the study coordinator during follow-up visits.

**Table 1:** Schedule of assessments

Questionnaire	Baseline	8 weeks	16 weeks	28 weeks	40 weeks	52 weeks
Informed Consent and Randomisation	X					
Demographic Information and Clinical Variable Questionnaire	X					
Seizure Diary	X	X	X	X	X	X
LAEP		X	X	X	X	X
HADS	X			X		X
EQ5D-5L	X			X		X
QOLIE-31	X			X		X
Healthcare Use Diary				X		X
WPAI-GH	X			X		X

EQ5D-5L, European Quality of Life 5 Dimensions 5 Level; HADS, Hospital Anxiety and Depression Scale; LAEP, Liverpool Adverse Events Profile; QOLIE-31, Quality of Life in Epilepsy-31; WPAI-GH, Work Productivity and Activity Impairment Questionnaire: General Health.

### Collection of saliva samples

The PICFs contain additional opt-in consent to the collection of a DNA sample for use in the current research only, future related research only, or any future research. Participants who opt in will be mailed a DNA Genotek OG-500^17^ self-administered saliva collection kit from the lead site with prepaid return postage. On return of the saliva sample, DNA extraction will be performed and the extracted DNA will be labelled according to the participants’ study number without any identifying information. The labelled DNA sample will be stored in a locked freezer at Monash University for future use.

### Participant safety and withdrawal

While this is a clinical trial involving the use of medication, the intervention in question is EpiMD rather than a new compound. Additionally, while for the ML group drug selection is guided by EpiMD, the ultimate decision lies with the neurologist. As such, the potential for an adverse reaction to the prescribed treatment beyond standard clinical care is low and is not expected to pose extra risk to participants. Adverse events will be systematically collected using the LAEP.^18^ Clinicians can enter free text to describe any adverse effects in the REDCap. The adverse reactions will be coded by standardised terminology according to the Medical Dictionary for Regulatory Activities.^19^ Participants are free to withdraw from the study at any time, for any reason, without prejudice. If a participant withdraws from the study early, their reason for early termination will be recorded in the study record.

### Primary outcomes

The primary outcome is the proportion of participants who achieve treatment success defined as seizure freedom while taking the prescribed ASM for the duration of the 12-month follow-up period. This outcome is consistent with the ILAE and European Medicines Agency guidelines for ASM monotherapy trials.^20^ This outcome is also clinically relevant given that seizure freedom is associated with improvement in quality of life in people with epilepsy.^2^

### Secondary outcomes

- Treatment failure is defined as withdrawal of the initial ASM or the commencement of polytherapy with a second ASM, whichever is earliest.
- Time to first seizure after treatment initiation.
- Direct healthcare costs will be assessed through review of a participant managed diary at both 28 weeks and 52-week post-treatment collection.
- Indirect healthcare costs will be assessed as a measure of productivity loss determined from the Work Productivity and Activity Impairment–General Health questionnaire, collected at baseline, 28 weeks and 52 weeks post-treatment commencement.
- The incidence of depression and anxiety will be assessed through changes to HADS scores^21^ over the treatment period, which will be collected at baseline, 28 weeks and 52 weeks post-treatment commencement.
- Comparison of the proportion of participants who achieve a minimum increase of 12 points in the Quality of Life in Epilepsy-31 (QOLIE-31) as a measure of quality of life,^22^ supported by the general European Quality of Life-5 Dimensions-5 Level (EQ-5D-5L) survey,^23^ both of which are collected at baseline, 28 weeks and 52 weeks post-treatment commencement.
- To assess clinical and participant acceptance of using ML to select the first ASM, participants and neurologists involved in the trial will be invited to take part in an optional process evaluation, involving:
  - A study-specific questionnaire developed based on the Unified Theory of Acceptance and Use of Technology 2.^24^
  - Qualitative interviews of 25% of study patients and 25% of study neurologists.

### Economic evaluation

Within-trial analysis of cost-effectiveness will compare differences in combined direct and indirect costs between the ML and usual care against differences for both quality-adjusted life-years and the 1-year seizure freedom rate. To ensure real-world relevance, prospective trial data will be analysed using the utility score indices from the EQ-5D-5L and QOLIE-31 overall score. This approach has been used in a previous health economic evaluation in epilepsy.^25^ If the results from this analysis indicate an increase in the seizure-free rate and quality of life, or a reduction in costs, extrapolation-based analysis over an extended time-horizon will be performed where costs and effects are expected to be discounted by the standard Australian 5% annual rate.

### Statistical analysis

Analysis of efficacy will be done for the intention-to-treat (ITT) set (all randomised patients who have taken at least one dose of the prescribed ASM) and the per-protocol set (patients in the ML group for whom neurologists did not prescribe the recommended drug will be excluded). The Cochran-Mantel-Haenszel test stratified by centres (to account for variation between centres) will compare the differences in 12-month seizure-free rate; retention rate (affected by both efficacy and tolerability) of the initial treatment at 1 year; and proportion of patients who achieve clinically meaningful improvement in quality of life. Cox regression will be used to assess differences in time to treatment failure and time to first seizure after treatment initiation between the two groups. Prescription patterns of the two groups will be compared. In the ML group, we will also compare the outcomes between patients who are prescribed the ML recommended ASMs and those who are prescribed non-ML recommended ASMs by their neurologists. This will include analysis of the reason provided by the neurologists in cases where ML recommendation was rejected. Also, the outcomes of different ASM groups such as sodium channel blockers versus non-sodium channel blockers will be compared.

Validation of EpiMD in the control group will be done by comparing the predicted response to the neurologist-selected ASM treatment outcome. A probability threshold of 0.55 will be applied in classifying treatment success or failure to calculate the model’s sensitivity, specificity and area under the curve.

To assess cost-effectiveness in the economic evaluation, cost differences and effect differences for 10 000 bootstrap draws will be analysed from the trial dataset over a 1-year time horizon. This will be represented using a cost-effective plane and a cost-effectiveness acceptability curve.^26^ This analysis will be performed both from the public healthcare and societal perspective. Consideration was given to the inclusion of an elderly cohort, particularly due to the prevalence of epilepsy in this age group. However, to match the current retirement age, we intend to maintain the upper age limit of 67. Based on the analytic results, future study directions such as extended follow-up duration will be considered, and given the distinct health and social needs of the older adult population,^27^ we propose the future development of a model specific to this cohort.

### Patient and public involvement

Members of the public have been involved since the design stage of this trial. Through a collaboration with the patient support organisation Epilepsy Action Australia, the lived experience expert (CS) was an associate investigator on the grant proposal and led the development of the trial consumer engagement strategy. This strategy involved forming a consumer committee comprised of people with epilepsy and is co-chaired by the lived experience expert and one of the trial investigators (EF). The committee will be responsible for ongoing review of the trial protocol and participant-facing trial documents. The Epilepsy Action Australia also facilitated interviews of patients with epilepsy. The result of these interviews indicated consumer support and acceptance for the use of AI in ASM selection.^28^

### Sample size

Based on previous RCTs in newly diagnosed epilepsy^29^,^32^ and observation in the Glasgow and Perth cohorts,^4 33^ the 12-month seizure-free rate on the first ASM in the usual care group is assumed to be 35% and to follow a distribution of Binomial (1000, 0.35) in participating sites. In keeping with ILAE recommendations for detecting clinically significant benefit,^34^ we hypothesise that the ITT 12-month seizure rate will be 20% higher (ie, 55%) when EpiMD is used. Allowing for non-compliance with the recommended drug by the investigators in 10% of patients in the ML group, with a one-sided significance level of 0.025 and 80% power, accounting for 10% dropout, a total of 234 participants (117 patients per group) will be recruited, determined from the 95th percentile of 1000 simulations. This will provide 90% power to detect a 23% higher ITT 1-year seizure rate in the ML group. This sample size will provide 80% power for the same minimal detectable effect size in retention rates and proportion of patients who report clinically meaningful improvement in QOLIE-31 total score. Additionally, this will provide for a minimum detectable HR of 0.58 in exit due to lack of efficacy, adverse events or any reason, and time to first seizure, assuming overall at least 50% event rates.

### Data security and handling

#### Clinical information

The clinical information is collected in this study during an initial interview, follow-up clinical visits, from data extracted from the EMR, text extraction from EEG and MRI reports, and from the results of questionnaires and assessments on the study schedule (see table 1). These data, along with all associated identifying information, will be held within the Monash University hosted REDCap Database. Within that database, an ‘identifying variable’ marker is applied to any identifying information. That information is paired with the participants assigned unique Study Record ID, when any data are exported from the REDCap server for analysis, information containing the identifying marker can be excluded from the export, allowing for curated management of the dataset and protection from identification. Any data (both identifying and non-identifying) contained within the REDCap Database is treated as sensitive health information and only members of the research team will be authorised with login credentials allowing them to view the data. A smaller group again will be authorised with write access to the database. Access to the research database will be audited using the individual login credentials provided. The principal and associate investigators from each site will only have access to identifying information from their own site as approved by their own site ethics and governance. The coordinating principal investigator and main site study coordinator will have access to all identifying information. Health information that has been collected will be kept for a minimum of 15 years. The PICFs contain additional opt-in consent items relating to retention of health information for future research. Data from participants who agree to these optional consent items will be retained indefinitely.

#### ML model

The code required to run EpiMD is hosted on a secure Australian Research Data Commons Nectar Research Cloud instance using Monash University access. Once all clinical variables required for the model have been entered in the REDCap survey, the investigator can follow a secure REDCap link that will initiate a run of the model and direct them back to the study REDCap where the model results can be found. After the processing time has completed, the recommendation will be sent to the REDCap result page. This page will display the results of the model and contain a text field to provide feedback about the decision-making surrounding the ASM recommendation (ie, whether the clinician chooses to prescribe the recommended ASM). This feedback, along with the individual score for each ASM assessed, will be retained for later analysis. For quality control and troubleshooting, an activity log of all variables and results that have been sent to and from the model is maintained on the model server.

#### Ethics and dissemination

This study has been granted multisite ethics approval by the Alfred Hospital HREC (Alfred HREC: 130/23) for all sites under National Mutual Acceptance (94107). Results from this study will be disseminated through publications in peer-reviewed journals and presentations at scientific conferences. Any amendments to the protocol will be approved by the relevant HREC prior to implementation. These changes will be reflected with an update to the ANZCTR registration (ANZCTR trial number: ACTRN12623000209695). We confirm that we have read the journal’s position on issues involved in ethical publication and affirm that this report is consistent with those guidelines. Findings will be presented in academic conferences and submitted to peer-reviewed journals for publication.

## Supplementary material

10.1136/bmjopen-2024-086607 online supplemental file 1

10.1136/bmjopen-2024-086607 online supplemental file 2
