# Supplementary material for: Personalised selection of medication for newly diagnosed adult epilepsy: study protocol of a first-in-class, double-blind, randomised controlled trial
Source: BMJ Open. 2025 Apr 5;15(4):e086607. doi: 10.1136/bmjopen-2024-086607 (PMC11973792; doi:10.1136/bmjopen-2024-086607)
Supplement: online supplemental file 1 [file bmjopen-15-4-s001.pdf]

Participant Information Sheet/Consent Form

Interventional Study - *Adult providing own consent*

Alfred Health

**Title**	Machine learning model for personalised drug selection in newly diagnosed epilepsy: a randomised controlled trial
**Short Title**	Personalised Selection of Medication for Newly Diagnosed Adult Epilepsy – the PERSONAL Trial
**Project Number**	ERM ID Number 94107
**Project Sponsor**	Monash University
**Coordinating Principal Investigator**	Professor Patrick Kwan
**Site Principal Investigator**	Prof Terence O'Brien
**Associate Investigator(s)**	Dr Richard Shek-Kwan Chang, Mr Daniel Thom, Prof Patrick Kwan, Dr Emma Foster, Dr Jonathan Baskin, Dr Merran Courtney, Dr Saba Mohidat, Dr Liz Edinburg Quiles, Dr Parv Sagar, Dr Andrew Neal, Dr John-Paul Nicolo Dr Noushin Chini Foroush, Dr Noam Bosak
**Location**	The Alfred Hospital

Part 1 What does my participation involve?

1 Introduction

You are invited to take part in this research project. This is because you have been diagnosed with epilepsy. This research project is testing a new method to select treatment for epilepsy. The new method is use of machine learning (a kind of artificial intelligence or AI) to recommend the first anti-seizure medication.

This Participant Information Sheet/Consent Form tells you about the research project. It explains the tests and treatments involved. Knowing what is involved will help you decide if you want to take part in the research.

Please read this information carefully. Ask questions about anything that you don't understand or want to know more about. Before deciding whether or not to take part, you might want to talk about it with a relative, friend or your local doctor.

Participation in this research is voluntary. If you don't wish to take part, you don't have to. You will receive the best possible care whether or not you take part.

If you decide you want to take part in the research project, you will be asked to sign the consent section. By signing it you are telling us that you:

• Understand what you have read

• Consent to take part in the research project
• Consent to have the tests and treatments that are described
• Consent to the use of your personal and health information as described.

You will be given a copy of this Participant Information and Consent Form to keep.

2 What is a machine learning (ML) model?

A ML model a is piece of software that takes data that has been collected to look for relationships between those pieces of data. Researchers can use an ML model to try and find connections between patient data such as prior medical history and predict how an individual might respond to different anti-seizure medications (ASM) for epilepsy. Using ML in this way might also assist in understanding why some people respond well to a certain anti-seizure medication, and why others do not.

3 What is the purpose of this research?

The ML model is an experimental method. This means that it is not an approved treatment for epilepsy in Australia. The purpose of this research is to find out if an ML model can be used to support your neurologist in choosing the first anti-seizure medication that should be prescribed for treatment of your epilepsy. The outcome of this research will show if a neurologist is better at selecting the most effective ASM when they have support from the ML model, or when they use only their own expert judgement.

The results of this research will also be used by the investigator Dr Richard-Shek-Kwan to obtain a PhD degree. This research has been initiated by the investigator, Professor Patrick Kwan.
This research has been funded by the National Health and Medical Research Council of Australia.

4 What does participation in this research involve?

After you provide consent to participate in the study, you will be asked questions about your epilepsy diagnosis and previous medical history. These questions are used to collect the data that will be used by the ML model.

You will be participating in a randomised controlled research project. Sometimes we do not know which treatment is best for treating a condition. To find out we need to compare different treatments. We put people into groups and give each group a different treatment. The results are compared to see if one is better. To try to make sure the groups are the same, each participant is put into a group by chance (random). You will either be placed into a group where the neurologist is told what ASM the machine learning model recommends or into a group where the neurologist is not told what ASM the machine learning model recommends.

You will have a one in two chance of having your ASM recommended by the machine learning model. Both groups will be followed for 12 months.

You will be participating in a double-blind study. This means that both you and the researcher or neurologist assessing your response to the prescribed ASM will not know which group you are randomised to. You and the researcher or neurologist assessing you will know the name and dose of the prescribed ASM. However, you and the researcher or neurologist assessing you will not know how it is chosen (i.e. with or without recommendation by the machine learning model). The purpose of this is to make sure the results will be interpreted in a fair and unbiased way.

There are no additional costs associated with participating in this research project, nor will you be paid. All medication, tests and medical care required as part of the research project will be provided to you free of charge.

You will have to pay for some medicines according to hospital policy. For example, dispensing fees for PBS-listed drugs.

If it is required, you will be reimbursed for the cost of second generation ASMs which are not covered in the Pharmaceutical Benefits Scheme for use as first-line treatment for epilepsy.

It is desirable that your local doctor be advised of your decision to participate in this research project. If you have a local doctor, we strongly recommend that you inform them of your participation in this research project.

You may be invited to participate in an optional sub-study related to this research

5 What do I have to do?

Once an anti-seizure medication has been prescribed by your neurologist you will be responsible for taking the medication according to the dosage and the schedule provided by your neurologist. The medication and dose may be adjusted as needed at subsequent clinic visits.

After you have started treatment you will be required to attend follow-up visits either in person or on telehealth with your neurologist at 8, 16, 28, 40 and 52 weeks, where you will be required to complete several surveys either online or on paper. This will take approximately 20-30 minutes. These surveys aim to collect information about your quality of life, anxiety and depression, your work productivity and healthcare costs.

If you consent to the optional collection of a DNA sample, you will be required to provide a saliva sample using a self-administered kit during your regular visits. This sample will be used for DNA extraction and storage in our biobank for future research.

While participating in this project you may be invited to take part in an optional sub-study aimed at explaining and interpreting the findings of this project. The sub-study will involve qualitative interview with researchers to answer questions related to satisfaction with the PERSONAL Trial, perceptions of support and suggestions for improvement.

6 Banking (Long term storage of samples)

"Banking" is storing health information and/or blood or tissue for future research studies. A "bank" is the place where the health information and/or blood or tissue is stored. Your DNA will be stored as re-identifiable/coded specimen(s).

The study doctor seeks your permission to store your DNA for future research. The study doctor would like you to consider taking part in this bank because you have epilepsy. In the future, other doctors and scientists at this and other medical and research centres may use your DNA to learn about many different diseases and conditions. Their goal is to improve health outcomes and develop new treatments.

The study doctor will store your sample/DNA at Monash University along with samples of many other people.

The purpose of storing your DNA in a bank is to answer questions in the future, so we expect to keep your DNA for a long time.

Your DNA will be stored at the Alfred Neuroscience Bio-databank. It will be stored as a re-identifiable sample. This means that your sample will be identifiable by a code; it can be identified as yours even though the bank does not know your identity. You can have it removed, destroyed or returned to you by contacting the study doctor, Professor Patrick Kwan, at Monash University, Central Clinical School, Alfred Centre, 99 Commercial Rd, 3004 VIC

7 What are the possible benefits of banking my DNA?

There is no direct benefit to you. Other people might benefit if researchers learn more by using your banked DNA.

8 What are the possible risks and disadvantages of banking?

Your sample/DNA will be stored in a re-identifiable form in the bank. Your sample/DNA will be stored securely in Monash University under an anonymous study number with no personal identifying information.

9 Other relevant information about the research project

Up to 234 people from fourteen hospitals will participate in this study. This research is a collaboration between doctors and researchers from The Alfred, Monash University, Royal Melbourne Hospital, Box Hill Hospital, Austin Hospital, Royal Brisbane and Women's Hospital, and Monash Medical Centre in Victoria; Royal Perth Hospital in Western Australia; Royal Prince Alfred Hospital, Prince of Wales Hospital, Westmead Hospital and Royal Northshore Hospital in New South Wales; Royal Adelaide Hospital and Queen Elizabeth Hospital in South Australia; and Royal Hobart Hospital in Tasmania.

This project also involves collaboration with researchers from Monash University.

10 Do I have to take part in this research project?

Participation in any research project is voluntary. If you do not wish to take part, you do not have to. If you decide to take part and later change your mind, you are free to withdraw from the project at any stage.

If you do decide to take part, you will be given this Participant Information and Consent Form to sign and you will be given a copy to keep.

Your decision whether to take part or not to take part, or to take part and then withdraw, will not affect your routine treatment, your relationship with those treating you or your relationship with Alfred Health.

11 What are the alternatives to participation?

You do not have to take part in this research project to receive treatment at this hospital. Other options are available; if you choose not to participate your neurologist will still be able to prescribe an anti-seizure medication without using the ML model. Your study doctor will discuss these options with you before you decide whether or not to take part in this research project. You can also discuss the options with your local doctor.

12 What are the possible benefits of taking part?

We cannot guarantee or promise that you will receive any benefits from this research; however, in some cases it takes some trial and error to select the most effective available anti-seizure medication. By participating in the study and using the ML model to support the neurologist's decision making, the most effective available anti-seizure medication may be selected earlier, which means that you could achieve freedom from seizures earlier than you would have without the ML model.

13 What are the possible risks and disadvantages of taking part?

Saliva Kit: There are no major risks associated with DNA collection using the saliva kit.

ML Model: The purpose of this research is to determine the effectiveness of the ML model in recommending an initial anti-seizure medication. As such it is possible that the study outcome will prove that the recommendation made with the model is not the most effective medication

and that a decision made only by the clinical expertise of the neurologist would have selected a more effective anti-seizure medication.

If you become upset or distressed as a result of your participation in the research, the study doctor will be able to arrange for counselling or other appropriate support. Any counselling or support will be provided by qualified staff who are not members of the research project team. This counselling will be provided free of charge.

Personal/Medical Information: This research project involves the collection of information about your use of illicit drugs. The test may reveal that you have previously used illegal drugs. That information will be stored in a re-identifiable (or coded) format. In the event that Alfred Health is required to disclose that information, it may be used against you in legal proceedings or otherwise.

14 What will happen to my test samples?

You will be asked to provide additional consent for the collection of your saliva during the research project.

Samples of your saliva obtained for the purpose of this research project will have the DNA extracted and stored in the Alfred Neuroscience Bio-databank at Monash University. The samples will be labelled only using a study identified number and without any personal identifying information. Only the study doctors for this study will have the code to reidentify you from the study ID. Your sample will not be sold by Monash University.

15 What if new information arises during this research project?

Sometimes during the course of a research project, new information becomes available about the treatment that is being studied. If this happens, your study doctor will tell you about it and discuss with you whether you want to continue in the research project. If you decide to withdraw, your study doctor will make arrangements for your regular health care to continue. If you decide to continue in the research project you will be asked to sign an updated consent form.

Also, on receiving new information, your study doctor might consider it to be in your best interests to withdraw you from the research project. If this happens, he/ she will explain the reasons and arrange for your regular health care to continue.

16 Can I have other treatments during this research project?

Whilst you are participating in this research project, you may not be able to take some or all of the medications or treatments you have been taking for your condition or for other reasons. It is important to tell your study doctor and the study staff about any treatments or medications you may be taking, including over-the-counter medications, vitamins or herbal remedies, acupuncture or other alternative treatments. You should also tell your study doctor about any changes to these during your participation in the research project. Your study doctor should also explain to you which treatments or medications need to be stopped for the time you are involved in the research project.

It may also be necessary for you to take medication during or after the research project to address side effects or symptoms that you may have. You may need to pay for these medications and so it is important that you ask your doctor about this possibility.

17 What if I withdraw from this research project?

If you decide to withdraw from the project, please notify a member of the research team when you withdraw. This notice will allow that person or the research supervisor to discuss any health risks or special requirements linked to withdrawing.

If you do withdraw your consent during the research project, the study doctor and relevant study staff will not collect additional personal information from you, although personal information already collected will be retained to ensure that the results of the research project can be measured properly and to comply with law. You should be aware that data collected by the sponsor up to the time you withdraw will form part of the research project results. If you do not want them to do this, you must tell them before you join the research project. If you have provided a saliva/DNA sample it will be disposed of when you withdraw.

18 Could this research project be stopped unexpectedly?

This research project may be stopped unexpectedly for a variety of reasons. These may include reasons such as:
• Unacceptable side effects
• The treatment being shown not to be effective
• The/treatment being shown to work and not need further testing

19 What happens when the research project ends?

Once the research project ends treatment for your epilepsy will continue to be managed by your neurologist as part of usual care.

Part 2 How is the research project being conducted?

20 What will happen to information about me?

By signing the consent form you consent to the study doctor and relevant research staff collecting and using personal information about you for the research project. Any information obtained in connection with this research project that can identify you will remain confidential. Your health information will be put into a controlled-access database housed at Monash University. This means that only researchers who apply for and get permission to use the information for a specific research project will be able to access the information. Researchers approved to access information in the database will agree not to attempt to identify you. Your information will only be used for the purpose of this research project and it will only be disclosed with your permission, except as required by law.

Information about you may be obtained from your health records held at this and other health services for the purpose of this research. By signing the consent form you agree to the study team accessing health records if they are relevant to your participation in this research project.

Your health records and any information obtained during the research project are subject to inspection (for the purpose of verifying the procedures and the data) by the relevant authorities and authorised representatives of the Sponsor, Monash University, the institution relevant to this Participant Information Sheet, Alfred Health, or as required by law. By signing the Consent Form, you authorise release of, or access to, this confidential information to the relevant study personnel and regulatory authorities as noted above.

It is anticipated that the results of this research project will be published and/or presented in a variety of forums. In any publication and/or presentation, information will be provided in such a way that you cannot be identified, except with your permission.

Information about your participation in this research project may be recorded in your health records.

In accordance with relevant Australian and Victorian privacy and other relevant laws, you have the right to request access to your information collected and stored by the research team. You also have the right to request that any information with which you disagree be corrected. Please

contact the study team member named at the end of this document if you would like to access your information.

Any information obtained for the purpose of this research project that can identify you will be treated as confidential and securely stored. It will be disclosed only with your permission, or as required by law.

If you do not consent to your information being used for future research it will be disposed of after 15 years which is the minimum retention period for data collected in a clinical trial.

21 What if I get injured in the research?

If you suffer any injuries or complications as a result of this research project, you should contact the study team as soon as possible and you will be assisted with arranging appropriate medical treatment. As you are eligible for Medicare, you can receive any medical treatment required to treat the injury or complication, free of charge, as a public patient in any Australian public hospital.

22 Who is organising and funding the research?

This research project is being conducted by Professor Patrick Kwan.

Monash University may benefit financially from this research project if, for example, the project assists Monash University to obtain approval to licence the ML model to use as part of usual management of epilepsy.

If knowledge acquired through this research leads to discoveries that are of commercial value to Monash University, the study doctors or their institutions, there will be no financial benefit to you or your family from these discoveries.

Alfred Health will receive a payment from Monash University for undertaking this research project.

No member of the research team will receive a personal financial benefit from your involvement in this research project (other than their ordinary wages).

23 Who has reviewed the research project?

All research in Australia involving humans is reviewed by an independent group of people called a Human Research Ethics Committee (HREC). The ethical aspects of this research project have been approved by the Alfred Hospital Ethics Committee

This project will be carried out according to the *National Statement on Ethical Conduct in Human Research (2007)*. This statement has been developed to protect the interests of people who agree to participate in human research studies.

24 Further information and who to contact

The person you may need to contact will depend on the nature of your query.

If you want any further information concerning this project or if you have any medical problems which may be related to your involvement in the project (for example, any side effects), you can contact the principal study doctor on 0450807412 or any of the following people:

Clinical contact person

Name	Mr Daniel Thom
Position	Study Coordinator
Telephone	0450807412

Email	d.thom@alfred.org.au

If you have any complaints about any aspect of the project, the way it is being conducted or any questions about being a research participant in general, then you may contact:

Reviewing HREC Office/Complaints contact person

Position	Complaints Officer, Office of Ethics & Research Governance, Alfred Health
Telephone	(03) 9076 3619
Email	research@alfred.org.au

Please quote the following Project ID number: 94107

Consent Form - *Adult providing own consent*

**Title**	Machine learning model for personalised drug selection in newly diagnosed epilepsy: a randomised controlled trial
**Short Title**	Personalised Selection of Medication for Newly Diagnosed Adult Epilepsy – the PERSONAL Trial
**Project Number**	ERM ID Number 94107
**Project Sponsor**	Monash University
**Coordinating Principal Investigator**	Professor Patrick Kwan
**Site Principal Investigator**	Professor Terence O'Brien
**Associate Investigator(s)**	Dr Richard Shek-Kwan Chang, Mr Daniel Thom, Prof Patrick Kwan, Dr Emma Foster, Dr Jonathan Baskin, Dr Merran Courtney, Dr Saba Mohidat, Dr Liz Quiles, Dr Parv Sagar, Dr Andrew Neal, Dr John-Paul Nicolo, Dr Noushin Chini Foroush, Dr Noam Bosak
**Location**	The Alfred Hospital

Consent Agreement

I have read the Participant Information Sheet or someone has read it to me in a language that I understand.

I understand the purposes, procedures and risks of the research described in the project.

I give permission for my doctors, other health professionals, hospitals or laboratories outside this hospital to release information to Monash University concerning my disease and treatment for the purposes of this project. I understand that such information will remain confidential.

I have had an opportunity to ask questions and I am satisfied with the answers I have received.

I freely agree to participate in this research project as described and understand that I am free to withdraw at any time during the study without affecting my future health care.

In respect to the collection and use of information, I give permission for use for the purpose of:

1. this specific research project Yes ☐ No ☐
2. other research that is closed related to this research project Yes ☐ No ☐
3. any future research Yes ☐ No ☐

I understand that I will be given a signed copy of this document to keep.

Declaration by Participant – for participants who have read the information

Name of Participant (please print) ______________________________

Signature ______________________ Date ______________________

Declaration - for participants unable to read the information and consent form
Witness to the informed consent process
Name (please print) ____

Signature _________________________ Date ________________________
 * Witness is not to be the Investigator, a member of the study team or their delegate. Witness must be 18 years or older.

Declaration by Study Doctor/Senior Researcher†

I have given a verbal explanation of the research project, its procedures and risks and I believe that the participant has understood that explanation.

Name of Study Doctor/
Senior Researcher† (please print) ______________________________________

Signature ________________________ Date ________________________

† A senior member of the research team must provide the explanation of, and information concerning, the research project.

Note: All parties signing the consent section must date their own signature.

I understand that, if I decide to discontinue the study treatment, I may be asked to attend follow-up visits to allow collection of information regarding my health status. Alternatively, a member of the research team may request my permission to obtain access to my medical records for collection of follow-up information for the purposes of research and analysis.

In respect to the storage and use of my saliva samples, I give permission for the use of DNA and/or tissue for the purpose of:

1. this specific research project Yes ☐ No ☐
2. other research that is closed related to this research project Yes ☐ No ☐
3. any future research Yes ☐ No ☐

In respect to future testing that may be performed with my DNA sample, I wish to be informed of any significant results that may be relevant to me Yes ☐ No ☐

By signing this consent section, I agree to the use of my saliva samples for genetic testing, as outlined in the relevant Section of the Participant Information Sheet.

Declaration by Participant – for participants who have read the information

Name of Participant (please print) ______________________________________

Signature ________________________ Date ________________________

For participants unable to read the information and consent form
Witness to the informed consent process
Name (please print) ____
Signature _________________________ Date ________________________
* Witness is not to be the Investigator, a member of the study team or their delegate. Witness must be 18 years or older.

Declaration by Study Doctor/Senior Researcher†
I have given a verbal explanation of the research project, its procedures and risks and I believe that the participant has understood that explanation.

Name of Study Doctor/ Senior Researcher† (please print)	____
Signature ____________________________	Date ____________________________

† A senior member of the research team must provide the explanation of and information concerning the research project.
Note: All parties signing the consent section must date their own signature.

If consent was obtained via telehealth or telephone please indicate with the following statements where appropriate:

- ❒ Consent was obtained using telehealth with *Participant:* _______________ whose photographic identification was sighted by the Investigator who observed the Participant's signature being written
- ❒ Consent was obtained via telephone with *Participant:*_______________ on ____/____/____.
- ❒ Participant's signed consent form received by the Investigator on ____/____/____.
- ❒ Consent was obtained using telehealth with *Investigator:* _____________ whose photographic identification was sighted by the Participant who observed the Investigator's signature being written
- ❒ Consent was obtained via telephone with *Investigator:*_____________ on ____/____/____.
- ❒ Discussed with *Participant:*__________ via telephone on ____/____/____ and received signed consent form on ____/____/____. Signed by *Investigator:*_____________

Form for Withdrawal of Participation - *Adult providing own consent*

**Title**	Machine learning model for personalised drug selection in newly diagnosed epilepsy: a randomised controlled trial
**Short Title**	Personalised Selection of Medication for Newly Diagnosed Adult Epilepsy – the PERSONAL Trial
**Project Number**	ERM ID Number 94107
**Project Sponsor**	Monash University
**Coordinating Principal Investigator**	Professor Patrick Kwan
**Site Principal Investigator**	Professor Terence O'Brien
**Associate Investigator(s)**	Dr Richard Shek-Kwan Chang, Mr Daniel Thom, Prof Patrick Kwan, Dr Emma Foster, Dr Jonathan Baskin, Dr Merran Courtney, Dr Saba Mohidat, Dr Liz Quiles, Dr Parv Sagar, Dr Andrew Neal, Dr John-Paul Nicolo, Dr Noushin Chini Foroush, Dr Noam Bosak
**Location**	The Alfred Hospital

Declaration by Participant

I wish to withdraw from participation in the above research project and understand that such withdrawal will not affect my routine treatment, my relationship with those treating me or my relationship with Alfred Health.

Name of Participant (please print) ______________________________

Signature ______________________ Date ______________________

Declaration by Study Doctor/Senior Researcher†

I have given a verbal explanation of the implications of withdrawal from the research project and I believe that the participant has understood that explanation.

Name of Study Doctor/
Senior Researcher† (please print) ______________________________

Signature ______________________ Date ______________________

† A senior member of the research team must provide the explanation of and information concerning withdrawal from the research project.

Note: All parties signing the consent section must date their own signature.
