# Supplementary material for: Personalised selection of medication for newly diagnosed adult epilepsy: study protocol of a first-in-class, double-blind, randomised controlled trial
Source: BMJ Open. 2025 Apr 5;15(4):e086607. doi: 10.1136/bmjopen-2024-086607 (PMC11973792; doi:10.1136/bmjopen-2024-086607)

**Supplemental Figure 1. Prediction of 1-year seizure freedom on first ASM for randomly selected participants.** The figure shows a prediction and rank of the likelihood of treatment success (seizure freedom at 12 months without intolerable adverse effects) of each of the seven predefined ASMs (which include the most used antiseizure medications in Australia) as the first monotherapy for each of 100 randomly selected participants from our pooled cohort.


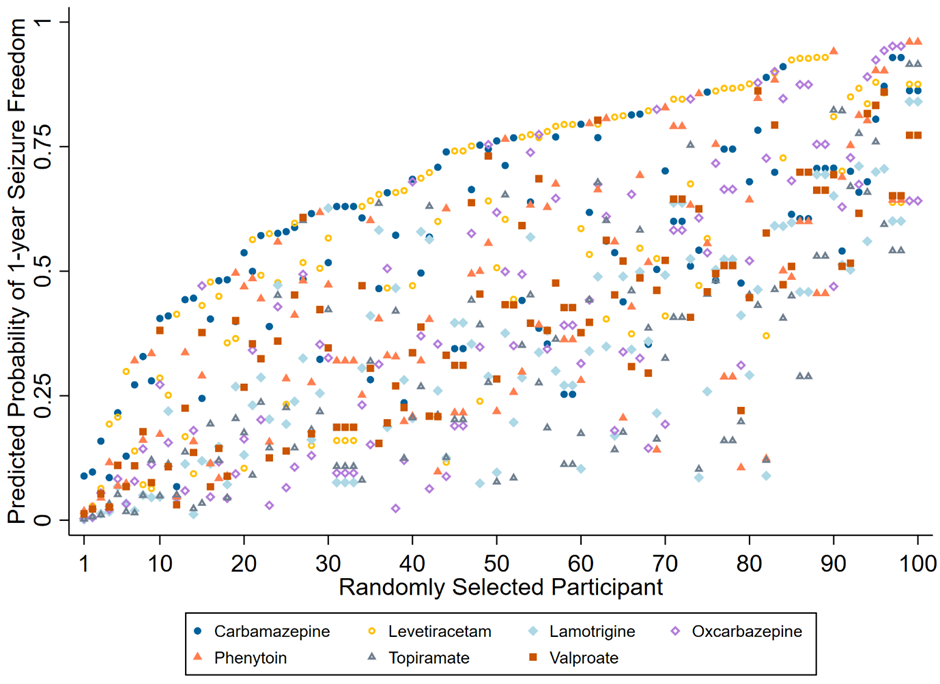

Supplement: online supplemental file 2 [file bmjopen-15-4-s002.docx]
