# Supplementary material for: Personalised selection of medication for newly diagnosed adult epilepsy: study protocol of a first-in-class, double-blind, randomised controlled trial
Source: BMJ Open. 2025 Apr 5;15(4):e086607. doi: 10.1136/bmjopen-2024-086607 (PMC11973792; doi:10.1136/bmjopen-2024-086607)
Supplement: Supplementary file 3 [file bmjopen-2024-086607.reviewer_comments.pdf]

PEER REVIEW HISTORY

BMJ Open publishes all reviews undertaken for accepted manuscripts. Reviewers are asked to complete a checklist review form and are provided with free text boxes to elaborate on their assessment. These free text comments are reproduced below.

ARTICLE DETAILS

Title (Provisional)

Personalised Selection of Medication for Newly Diagnosed Adult Epilepsy: study protocol of a first-in-class, double-blind, randomised controlled trial

Authors

Thom, Daniel; Chang, Richard Shek-kwan; Lannin, Natasha A; Ademi , Zanfina; Ge, Zongyuan; Reutens, David; O'Brien , Terence; D'Souza, Wendyl; Perucca, Piero; Reeder, Sandra; Nikpour, Armin; Wong, Chong; Kiley, Michelle; Saw, Jacqui-Lyn; Nicolo, John-Paul; Seneviratne, Udaya; Carney, Patrick; Jones, Dean; Somerville, Ernest; Stapleton, Clare; Foster, Emma; Vadlamudi, Lata; Vaughan, David N.; Lee, James; Farrar, Tania; Howard, Mark; Sparrow, Robert; Chen, Zhibin; Kwan, Patrick

---

VERSION 1 - REVIEW

**Reviewer**	**1**
**Name**	**Yao, Lijun**
**Affiliation**	**Tongji University School of Medicine**
**Date**	**17-May-2024**
**COI**	**No**

The research protocol addresses a significant clinical challenge in epilepsy management: selecting appropriate antiseizure medication (ASM) for newly diagnosed patients. The use of machine learning to personalize treatment is a promising approach and aligns with current trends in precision medicine." However, some issues still need further clarification in the draft.

1. The double-blind, randomized controlled trial design is robust and appropriate for evaluating the efficacy of the ML model. However, the exclusion criteria are quite restrictive, which may limit the generalizability of the results. It is crucial for the authors to delve into how the results might apply to a broader patient population, thereby enhancing the potential impact of the study.

2. It is highly recommended that an in-depth analysis of the difference between the ASM recommended by the model and the ASM selected by the clinician be added to the study.

This will significantly contribute to a better understanding of the role of ML models in actual clinical decision-making, thereby enriching the study's findings.

3. The number of ASMs selected in the study is limited (seven types), which may not fully represent the clinically available ASMs, affecting the flexibility and applicability of model recommendations.

4. The study's primary outcome measure is the 12-month seizure-free, but the evaluation of long-term treatment effects and safety may need to be revised.

**Reviewer**	**2**
**Name**	**Ricci, Lorenzo**
**Affiliation**	**Università Campus Bio-Medico di Roma**
**Date**	**10-Jun-2024**
**COI**	**I have no competing interests to declare.**

The manuscript proposes a protocol for a first-in-class, double-blind, randomized controlled trial, aiming to develop an algorithm for personalized anti-seizure medication (ASM) selection in individuals with epilepsy. The manuscript is of extreme interest to me; the scientific background is well-written and thoroughly discussed, and the methodology is appropriate and scientifically robust. The sample size is adequate. I have a few minor concerns (listed below, not in order of importance):

A 52-week follow-up may not be sufficient if seizure frequency is more sporadic. The authors may consider assessing a secondary objective: a 2-year clinical follow-up, if feasible.

The authors may consider including a secondary analysis based on the mechanism of action of the drugs (e.g., sodium channel blockers) rather than the individual molecules.

Integrating a sleep EEG report into the model could be extremely beneficial. The authors should consider adding this variable to the revised protocol.

The algorithm will not be validated for elderly people (over 65 years old), who are an especially important population given the prevalence of epilepsy in this age group. A sub-analysis for this particular age group could be considered, limiting the choice of ASMs to those with a better safety profile for the elderly.

There is a minor concern regarding the choice of ASM in people with epilepsy and other comorbidities that may already guide toward an optimal ASM selection (e.g., Topiramate in patients with comorbid migraines, Valproic Acid for patients with mood disorders). How do the authors intend to ethically account for this fact, since individuals in the algorithm arm may not receive optimal therapy for their conditions?

It would be interesting to identify the features utilized by the model for predicting the ASM selection, which may not be feasible given the nature of the model. Additionally, I have a concern regarding the inclusion of both focal and generalized epilepsy in the same trial. This inclusion may affect the actual applicability of the algorithm since these conditions usually require a very different approach in terms of ASM selection. This is also true for other specific etiology of epilepsy (i.e., metabolic, certain genetic causes; Kearney, Hugh, et al. "Tackling epilepsy with high-definition precision medicine: a review." JAMA Neurology 76.9 (2019): 1109-1116.). I suggest performing a sub-analysis dividing these populations (or at least focal epilepsy and generalized/genetic epilepsies).

VERSION 1 - AUTHOR RESPONSE

Reviewer: 1

Dr. Lijun Yao, Tongji University School of Medicine

The research protocol addresses a significant clinical challenge in epilepsy management: selecting appropriate antiseizure medication (ASM) for newly diagnosed patients. The use of machine learning to personalize treatment is a promising approach and aligns with current trends in precision medicine." However, some issues still need further clarification in the draft.

1. The double-blind, randomized controlled trial design is robust and appropriate for evaluating the efficacy of the ML model. However, the exclusion criteria are quite restrictive, which may limit the generalizability of the results. It is crucial for the authors to delve into how the results might apply to a broader patient population, thereby enhancing the potential impact of the study.

Response: Our exclusion criteria are similar to other antiseizure medication trials.[1,2] However, we acknowledge that the current criteria will exclude certain important subgroups. It can be a direction of further studies. The following changes have been made to the “Statistical Analysis” section (page 17, lines 11-16):

“Consideration was given to inclusion of an elderly cohort, particularly due to the prevalence of epilepsy in this age group. However, to match the current retirement age we intend to maintain the upper age limit of 67. Based on the analytic results, future study directions such as extended follow-up duration will be considered and given the distinct health and social needs of the older adult population [5], we propose the future development of a model specific to this cohort.”

2. It is highly recommended that an in-depth analysis of the difference between the ASM recommended by the model and the ASM selected by the clinician be added to the study. This will significantly contribute to a better understanding of the role of ML models in actual clinical decision-making, thereby enriching the study's findings.

Response: Thank you for the helpful suggestion. We have added the following sentences in the “Statistical analysis” section (page 16, line 18-21):

“Prescription patterns of the two groups will be compared. In the ML group, we will also compare the outcomes between patients who are prescribed the ML recommended ASMs and those who are prescribed non-ML recommended ASMs.”

3. The number of ASMs selected in the study is limited (seven types), which may not fully represent the clinically available ASMs, affecting the flexibility and applicability of model recommendations.

Response: The model currently used is developed from an earlier published version.[3] That model selects one out of the seven drugs for the newly diagnosed epilepsy patients similar to current model. These seven drugs included the most commonly used antiseizure medications in Australia.[4] Nonetheless, we agree that the model should be updated in the future in line with changes in prescribing practice. The following sentence has been added to the introduction section (page 7, lines 12-13):

“seven ASMs (which include the most used antiseizure medications in Australia)”

Additionally, the following sentence has been added to the limitations section (page 5, line 17-19):

“The trial confines the choice of drug to seven commonly prescribed antiseizure medications limiting the flexibility of neurologists who may have prescription preference outside the repertoire”

4. The study's primary outcome measure is the 12-month seizure-free, but the evaluation of long-term treatment effects and safety may need to be revised.

Response: We acknowledge the 12-month duration of study as a limitation and will consider extended follow-up time depending on the available resources. We have added the sentence under the “Statistical analysis” (Page 17, lines 13-14):

“Based on the analytic results, future study directions such as extended follow-up duration will be considered.”

Reviewer: 2

Dr. Lorenzo Ricci, Università Campus Bio-Medico di Roma

Comments to the Author:

The manuscript proposes a protocol for a first-in-class, double-blind, randomized controlled trial, aiming to develop an algorithm for personalized anti-seizure medication (ASM) selection in individuals with epilepsy. The manuscript is of extreme interest to me; the scientific background is well-written and thoroughly discussed, and the methodology is appropriate and scientifically robust. The sample size is adequate. I have a few minor concerns (listed below, not in order of importance):

A 52-week follow-up may not be sufficient if seizure frequency is more sporadic. The authors may consider assessing a secondary objective: a 2-year clinical follow-up, if feasible.

Response: We agree that a longer follow-up period should be considered. We have added the sentence under the “Statistical analysis” (page 17, lines 13-14):

“Based on the results, future study directions such as extended follow-up duration will be considered.”

The authors may consider including a secondary analysis based on the mechanism of action of the drugs (e.g., sodium channel blockers) rather than the individual molecules.

Response: Thank you for the suggestion. We have added the following sentence under the “Statistical analysis” (page 17, lines 13-14):

“Prescription patterns of the two groups will be compared.”

We have also added the following sentence under the “Statistical analysis” (page 16, lines 22-24):

“Also, the outcomes of different ASM groups such as sodium channel blockers versus non-sodium channel blockers will be compared.”

Integrating a sleep EEG report into the model could be extremely beneficial. The authors should consider adding this variable to the revised protocol.

Response: Thank you for the suggestion. For pragmatic reason routine EEG has been included as an eligibility criterion but not sleep EEG. Nonetheless, sleep EEG report will be analysed if it has been performed as clinically dictated. The following changes have been made to the introduction section (page 7, line 19-21):

“Consideration was made to the use of sleep EEG reports, while they are not precluded from use in place of a routine EEG, for pragmatic reasons they are not a mandatory input.”

Additionally, the following change has been made to the Methods section (page 10, lines (4-6):

“Routine EEG and MRI brain done within 2 years months prior to randomisation is also required. The routine EEG can (but is not specifically required to) be replaced with a sleep EEG.”

The algorithm will not be validated for elderly people (over 65 years old), who are an especially important population given the prevalence of epilepsy in this age group. A sub-analysis for this particular age group could be considered, limiting the choice of ASMs to those with a better safety profile for the elderly.

Response: The choice of the upper age limit (revised to 67 years to reflect the recent change in retirement age in Australia) is related to the health economic analysis currently embedded in the trial. Given the distinct health and social needs (Dwyer et al, 2023), we propose that a separate model should be developed in the future for the older adult population. The following changes have been made to the “Statistical Analysis” section (page 17, lines 11-16):

“Consideration was given to inclusion of an elderly cohort, particularly due to the prevalence of epilepsy in this age group. However, to match the current retirement age we intend to maintain the upper age limit of 67. Based on the analytic results, future study directions such as extended follow-up duration will be considered and given the distinct health and social needs of the older adult population (Dwyer et al, 2023), we propose the future development of a model specific to this cohort.”

Additionally, the limitation “Participants will be recruited from tertiary epilepsy centres, results may not be generalised to all those with newly diagnosed epilepsy” has been replace with the following: (page 5, lines 20-22)

“The inclusion criteria of aged 18-67 specifically includes the older adult epilepsy cohort how have a high prevalence of epilepsy along with distinct health and social needs that could be alleviated by optimised initial ASM selection”

There is a minor concern regarding the choice of ASM in people with epilepsy and other comorbidities that may already guide toward an optimal ASM selection (e.g., Topiramate in patients with comorbid migraines, Valproic Acid for patients with mood disorders). How do the authors intend to ethically account for this fact, since individuals in the algorithm arm may not receive optimal therapy for their conditions?

Response: This has been ethically accounted for given that the neurologist responsible for randomizing a patient is able to reject the recommendation of the model because of safety concern or other reasons. Reasons provided by the neurologists in case of rejection of the ML recommendation will be analysed. The following changes have been made to the “Statistical analysis section (page 16, lines 21-22):

“, this will also include analysis of the reason provided by the neurologist in cases where the ML recommendation has been rejected.”

It would be interesting to identify the features utilized by the model for predicting the ASM selection, which may not be feasible given the nature of the model. Additionally, I have a concern regarding the inclusion of both focal and generalized epilepsy in the same trial. This inclusion may affect the actual applicability of the algorithm since these conditions usually require a very different approach in terms of ASM selection. This is also true for other

specific etiology of epilepsy (i.e., metabolic, certain genetic causes; Kearney, Hugh, et al. "Tackling epilepsy with high-definition precision medicine: a review." JAMA Neurology 76.9 (2019): 1109-1116.). I suggest performing a sub-analysis dividing these populations (or at least focal epilepsy and generalized/genetic epilepsies).

Response: We acknowledge that the drug responsiveness of generalized epilepsy is different from focal epilepsy. Hence, one of the features of our model is whether the epilepsy is focal or non-focal. We will also perform subgroup analysis based on the type of epilepsy. We do not plan to analyse specific aetiologies given that the number of patients with each aetiology will likely be small. The following changes have been made to the limitations section (page 6, lines 1-3):

"The model is utilised both on patients with generalised epilepsy or focal epilepsy, planned subgroup analysis is limited to these two epilepsy types and does not include more specific aetiologies."

VERSION 2 - REVIEW

**Reviewer**	**1**
**Name**	**Yao, Lijun**
**Affiliation**	**Tongji University School of Medicine**
**Date**	**18-Sep-2024**
**COI**	**no**

---

1. The ML model previously established by the authors was designed to predict the outcomes after drug treatment, not the selection of drugs. Before applying the model to clinical trials, it should be tested using existing data on how much difference the model predicts if one of the seven drugs is used respectively.

2. The established model's data volume is relatively large, but it comes from five different datasets and four countries, and the AUROC value reflecting the predictive effect is also low (0.65). There are inherent differences in the characteristics of populations from different regions and treatment choices. Before clinical application, re-optimizing the model with locally collected data might yield better results.

3. It is recommended that the differences between the preferred drugs given by the model and those given by neurologists be compared.

VERSION 2 - AUTHOR RESPONSE

Reviewer: 1
Dr. Lijun Yao, Tongji University School of Medicine Comments to the Author:

Q1.

The ML model previously established by the authors was designed to predict the outcomes after drug treatment, not the selection of drugs. Before applying the model to clinical trials, it should be tested using existing data on how much difference the model predicts if one of the seven drugs is used respectively.

Response:

Thank you for your comment. Our ML model is designed to predict outcomes to individual ASM based on participants' clinical features prior to commencing treatment. Testing the model using existing data to evaluate absolute differences in seizure-free rates between drugs would require dichotomising these probabilities into seizure freedom outcomes. This process would necessitate applying an arbitrary cut-off, which we believe would be inappropriate and could misrepresent the model's purpose. Instead, the model's strength lies in its ability to rank treatment options for individualised clinical decision-making, which will be validated in the current clinical trial.

We have included a new supplementary figure to illustrate this, and made the following changes in the Introduction section (paragraph 3):

" It was trained to predict and rank the likelihood of treatment success (seizure freedom at 12 months without intolerable adverse effects) of each of the seven predefined ASMs (which include the most used antiseizure medications in Australia) as the first monotherapy for an individual patient. This ability is illustrated in 100 individuals randomly selected from the pooled cohort in Supplementary figure."

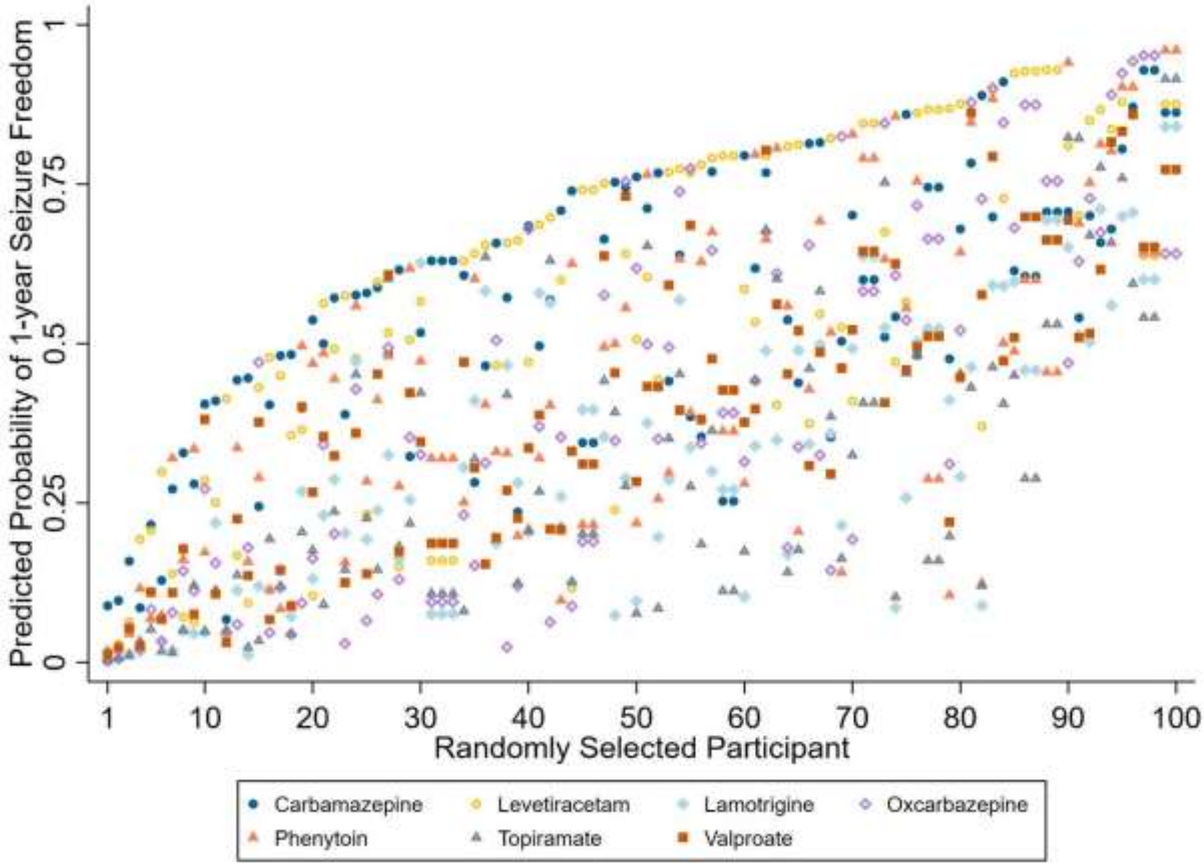

Figure 1. Predicted probability of 1-year seizure freedom on first ASM for randomly selected population of 100 participants

Q2:

The established model's data volume is relatively large, but it comes from five different datasets and four countries, and the AUROC value reflecting the predictive effect is also low (0.65). There are inherent differences in the characteristics of populations from different regions and treatment choices. Before clinical application, re-optimizing the model with locally collected data might yield better results.

Response 2:

The AUROC value of 0.65 refers to the original model published in JAMA Neurology (Hakeem 2022). As described in the Introduction, we have improved this model by applying natural language processing to extract information from the text of EEG and MRI brain reports. The improved model was re-optimized using datasets from Australian and international cohorts. Ultimately, the effectiveness of applying such a model in clinical practice would require prospective evaluation, which is why the current study is being undertaken.

The following changes have been made to the Introduction section (paragraph 4):

"The improved model was re-optimized using datasets from Australian and international cohorts. Ultimately, the effectiveness of applying such a model in clinical practice would require prospective evaluation."

Q3:

It is recommended that the differences between the preferred drugs given by the model and those given by neurologists be compared.

Response 3:

Thank you for this suggestion. As described in the Statistical Analysis section (paragraph 1), "in the ML group we will also compare the outcomes between patients who are prescribed the ML recommended ASMs and those who are prescribed non-ML recommended ASMs by their neurologists. This will include analysis of the reason provided by the neurologists in cases where ML recommendation was rejected".

VERSION 3 - REVIEW

**Reviewer**	**1**
**Name**	**Yao, Lijun**
**Affiliation**	**Tongji University School of Medicine**
**Date**	**20-Jan-2025**
**COI**	

The authors have answered all of my questions.
